# Supplementary material for: Effect of ethnicity and other sociodemographic factors on attendance at diabetic eye screening: a 12-month retrospective cohort study
Source: BMJ Open. 2021 Sep 17;11(9):e046264. doi: 10.1136/bmjopen-2020-046264 (PMC8451288; doi:10.1136/bmjopen-2020-046264)
Supplement: Author's manuscript [file bmjopen-2020-046264.draft_revisions.pdf]

BMJ Open is committed to open peer review. As part of this commitment we make the peer review history of every article we publish publicly available.

When an article is published we post the peer reviewers' comments and the authors' responses online. We also post the versions of the paper that were used during peer review. These are the versions that the peer review comments apply to.

The versions of the paper that follow are the versions that were submitted during the peer review process. They are not the versions of record or the final published versions. They should not be cited or distributed as the published version of this manuscript.

BMJ Open is an open access journal and the full, final, typeset and author-corrected version of record of the manuscript is available on our site with no access controls, subscription charges or pay-per-view fees (http://bmjopen.bmj.com).

If you have any questions on BMJ Open's open peer review process please email info.bmjopen@bmj.com

BMJ Open

Sociodemographic inequalities in diabetic eye screening attendance among a large multi-ethnic inner-city population with high levels of deprivation

Journal:	*BMJ Open*
Manuscript ID	bmjopen-2020-046264
Article Type:	Original research
Date Submitted by the Author:	16-Dec-2020
Complete List of Authors:	Olvera-Barrios, Abraham; Moorfields Eye Hospital NHS Foundation Trust, Medical Retina; University College London Institute of Ophthalmology<br>Seltene, Michael; Moorfields Eye Hospital NHS Foundation Trust, Diabetic Retinal Screening<br>Heeren, Tjebo; Moorfields Eye Hospital NHS Foundation Trust, Medical Retina; University College London Institute of Ophthalmology<br>Chambers, Ryan; Homerton University Hospital NHS Foundation Trust<br>Bolter, Louis; Homerton University Hospital NHS Foundation Trust<br>Tufail, Adnan; Moorfields Eye Hospital NHS Foundation Trust, ; UCL Institute of Ophthmology,<br>Owen, C; St George's, University London, Population Health Research Institute<br>Rudnicka, Alicja; St George's University of London, Population Health Research Institute<br>Egan, Catherine; Moorfields Eye Hospital; University College London Institute of Ophthalmology<br>Anderson, John; Homerton University Hospital NHS Foundation Trust
Keywords:	Diabetic retinopathy < DIABETES & ENDOCRINOLOGY, EPIDEMIOLOGY, SOCIAL MEDICINE, Clinical audit < HEALTH SERVICES ADMINISTRATION & MANAGEMENT, Quality in health care < HEALTH SERVICES ADMINISTRATION & MANAGEMENT

SCHOLARONE™
Manuscripts

I, the Submitting Author has the right to grant and does grant on behalf of all authors of the Work (as defined in the below author licence), an exclusive licence and/or a non-exclusive licence for contributions from authors who are: i) UK Crown employees; ii) where BMJ has agreed a CC-BY licence shall apply, and/or iii) in accordance with the terms applicable for US Federal Government officers or employees acting as part of their official duties; on a worldwide, perpetual, irrevocable, royalty-free basis to BMJ Publishing Group Ltd ("BMJ") its licensees and where the relevant Journal is co-owned by BMJ to the co-owners of the Journal, to publish the Work in this journal and any other BMJ products and to exploit all rights, as set out in our licence.

The Submitting Author accepts and understands that any supply made under these terms is made by BMJ to the Submitting Author unless you are acting as an employee on behalf of your employer or a postgraduate student of an affiliated institution which is paying any applicable article publishing charge ("APC") for Open Access articles. Where the Submitting Author wishes to make the Work available on an Open Access basis (and intends to pay the relevant APC), the terms of reuse of such Open Access shall be governed by a Creative Commons licence – details of these licences and which Creative Commons licence will apply to this Work are set out in our licence referred to above.

Other than as permitted in any relevant BMJ Author's Self Archiving Policies, I confirm this Work has not been accepted for publication elsewhere, is not being considered for publication elsewhere and does not duplicate material already published. I confirm all authors consent to publication of this Work and authorise the granting of this licence.

For peer review only

Sociodemographic inequalities in diabetic eye screening attendance among a large multi-ethnic inner-city population with high levels of deprivation

Abraham Olvera-Barrios,[1-2] Michael Seltene,[3] Tjebo F.C. Heeren,[1-2] Ryan Chambers,[4] Louis Bolter,[4] Adnan Tufail,[1-2] Christopher G. Owen,[5] Alicja R. Rudnicka,[5] Catherine A. Egan,[1-2] John Anderson.[4]

1. Medical Retina, Moorfields Eye Hospital NHS Foundation Trust, London, United Kingdom
2. University College London Institute of Ophthalmology, London, United Kingdom
3. Diabetic Retinopathy Screening, Moorfields Eye Hospital NHS Foundation Trust, London, United Kingdom
4. Diabetes, Homerton University Hospital NHS Foundation Trust, London, United Kingdom
5. Population Health Research Institute, St George's University of London, London, United Kingdom

Corresponding Author
John Anderson
Homerton University Hospital NHS Foundation Trust
john.anderson@nhs.net

Word count: 2814

For peer review only - http://bmjopen.bmj.com/site/about/guidelines.xhtml

ABSTRACT

Objectives: To examine the association of sociodemographic characteristics with attendance at diabetic eye screening in a large ethnically diverse urban population.

Design: Retrospective cohort study.

Setting: Screening visits in the North East London Diabetic Eye Screening Programme (NELDESP).

Participants: 84,449 people with diabetes aged 12 years or older registered in the NELDESP and scheduled for screening between 1st April 2017 to 31st March 2018.

Main outcome measure: Association between sociodemographic factors (age, gender, self-defined ethnicity, area level deprivation), type of diabetes, duration of diabetes, visual acuity, years of NELDESP registration, distance to screening centre, and Public Transport Accessibility, with attendance for diabetic eye screening.

Results: The mean age of people with diabetes was 60 yrs (SD 14.2 yrs), 53.4% were males, 41% South Asian, 29% White British and 17% Black; 83.4 % attended screening. Black people with diabetes had similar levels of attendance compared with White British people. However, South Asian, Chinese and any other Asian background ethnicities showed greater odds of attendance compared with White British. When compared with their respective reference group, high levels of deprivation, younger age, longer duration of diabetes, worse visual acuity and longer distance to screening centre, were all associated with non-attendance. There was a higher likelihood of attendance per quintile improvement in deprivation (odds ratio [OR], 1.06; 95%CI, 1.03-1.08), with increasing age (OR per decade, 1.17; 95%CI, 1.15-1.19), with better visual acuity (OR per Bailey-Lovey chart line 1.12, 95%CI 1.11-1.14) and with longer time of NELDESP registration (OR per 5 yrs, 1.12; 95%CI, 1.08-1.17).

Conclusion: Ethnic differences in diabetic eye screening uptake are evident, but despite preconceptions a higher likelihood of screening attendance was observed among Asians compared with whites. Poorer socioeconomic profile was associated with higher likelihood of non-attendance for screening. Further work is needed to understand how to target individuals at risk of non-attendance and reduce inequalities.

For peer review only - http://bmjopen.bmj.com/site/about/guidelines.xhtml

Article summary

Strengths and Limitations of the study

- We used a retrospective cohort of 84,449 people to address a key issue in diabetic retinopathy screening: The association of sociodemographic factors with non-attendance to a systematic diabetic eye screening programme.
- Strengths contain that our study is one of the most current analysis with high-quality data on ethnicity, a diverse population with high socioeconomic deprivation, and the inclusion of additional factors, such as, the distance to screening centre and public transport accessibility level.
- Systemic risk factors for diabetic retinopathy incidence and progression, and the association of sociodemographic variables with diabetic retinopathy were not available to analyse.
- Our study cohort is from people with health coverage registered in a systematic diabetic eye screening programme, hence results cannot be extrapolated to populations from different settings.

For peer review only

INTRODUCTION

Diabetic retinopathy is a common neurovascular complication of diabetes and a major cause of blindness.(1, 2) There are at least 3.9 million people diagnosed with diabetes in the United Kingdom, a number expected to rise to 5.8 million by 2025.(3) It is estimated that 30% of people with diabetes will develop retinopathy, and about 9% will develop sight-threatening retinopathy.(4) An early diagnosis through population screening, timely referral and treatment are essential for prevention of diabetes-related visual impairment.(5-7) The UK implemented the first systematically organised diabetic eye screening programme (DESP) in the world in England in 2003, achieving nation-wide coverage by 2008. The English DESP offers annual mydriatic photographic screening to all people with diabetes aged 12 or older.(7) In accordance with national standards, screening of ≥85% of the eligible diabetic population is considered achievable, however, English DESP uptake data from 2016-2017 showed that this was not met in 75% of London's Clinical Commissioning Groups (CCGs) areas.(8, 9) Regional differences in screening delivery and uptake may explain regional variation in diabetic eye disease.(4)

Non-attendance at annual diabetic eye screening visits has been associated with late presentation of sight-threatening retinopathy.(10, 11) Inequalities in health tend to be present in urban areas with contrasting sociodemographic conditions. London, a metropolis where people from the extremes of the deprivation indices live side-by-side, is a remarkable example of how these inequalities can result in different uptake rates across and within boroughs.(12-14) Health inequalities can create significant attendance variation among subgroups, and are of concern to any screening programme. Sociodemographic factors such as, age (15-22), gender,(22-24) ethnicity,(15, 16, 24) transportation,(25) and socioeconomic deprivation (15-17, 20-22, 24, 26, 27) have all been associated with non-attendance.

The North East London population is sociodemographically diverse, with a wide variation in ethnicities and a varied health profile with higher than average level of deprivation and a lower than average life expectancy.(28-30) The North East London DESP (NELDESP) serves a total eligible population of approximately 125 000 people with diabetes aged 12 and over.(28) The NELDESP aims to invite ≥98% of eligible individuals and to have an uptake ≥85%. We examined the sociodemographic determinants of attendance at the NELDESP, within this multi-ethnic population with high levels of deprivation.

For peer review only

For peer review only - http://bmjopen.bmj.com/site/about/guidelines.xhtml

METHODS

We performed a 12-month retrospective cohort study between 1st April 2017 to 31st March 2018. The study was registered as an audit and approved by the Homerton University Hospital NHS Foundation Trust Research and Innovation Department.

Setting

The NELDESP is provided by the Homerton University Hospital. We analysed data from people with diabetes living in 6 CCG areas with inner city multi-ethnic populations, residing in London boroughs of Newham, Redbridge, Tower Hamlets and Waltham Forest, which have been classified as the most ethnically diverse in London;(31, 32) Barking & Dagenham and Hackney, which have a substantial multi-ethnic population.

The NELDESP is run according to English DESP guidelines. All people with diabetes aged 12 or older are identified through coding in primary care electronic record systems. Software is used to generate invitations to attend for NELDESP appointments. The Homerton Hospital carries out appointment call/recall, screening, image grading, referral tasks, and is responsible for providing clinical leadership and programme management, including failsafe procedures and internal quality assurance.(28)

Briefly, a screening visit entails a visual acuity assessment, and pupil dilation to obtain two 45° digital retinal images of each eye, centred on the fovea and disc, respectively. We have described in detail the imaging, grading protocol and referral pathway elsewhere.(33)

Data extraction

We carried out an anonymised data extraction of all screening appointments between the study period using structure query language (SQL) searches. An anonymised data base for analysis was created.

Independent variable recording

Ethnicity

Self-classified ethnicity data was collected from patients at the time of screening, or from the routinely recorded ethnicity data provided by their GP surgery. Their ethnicity was recorded in the nationally mandated screening software in accordance with the 2011 Office for National Statistics census groups.(31)

Index of multiple deprivation (IMD)

The English indices of deprivation are composed of 39 indicators arranged in 7 different domains of deprivation, which are combined and weighted to create the IMD, the official measure of relative deprivation in England. This measure is calculated for every neighbourhood or small area (lower-layer super output area [LSOA]) in England. There are 32,844 LSOAs with an average population of 1,500, and each of them is ranked from 1st, the most deprived area, to 32, the 844th

For peer review only - http://bmjopen.bmj.com/site/about/guidelines.xhtml

least deprived area. Patient's postcodes were linked to their LSOA indices of multiple deprivation scores.

Visual acuity, distance and Public Transport Accessibility Level (PTAL)

We recorded the most recent visual acuity within a 3-year time frame in Snellen notation for the analysis. The better-seeing eye visual acuity score was assigned to each person. We calculated distance to screening centre (in kilometres) as a straight line from the patient's postcode to the destination. The PTAL is a metric tool from Transport for London which rates locations by distance to the public transport network, thus reflecting the accessibility to public transport within Greater London. The PTAL grade takes into account walk access time, average waiting time, service availability and service reliability. The grading has 9 levels from 0 (with the poorest access) to 6b (excellent access).(34) Using Transport for London's Web-based Connectivity Assessment Toolkit (WebCAT),(35) we extracted the PTALs for each patient's home postcode.

Statistical analysis

We used R version 4.0.0 for statistical analysis.(36) We conducted a multivariable logistic regression analysis of attendance at screening visit (binary outcome coded "1" if patient attended and "0" if they did not attend). A test for trend was performed if the odds ratios showed a linear pattern across categorical variables. Attendance was defined as a participant completing the diabetic retinopathy screening process. Independent variables considered were age, gender, ethnicity, IMD, type of diabetes, duration of diabetes, visual acuity, years of registration into the DESP, distance to screening centre, and PTAL.

We categorised continuous variables for the analysis to allow for non-linear patterns in attendance. Rank scores of the IMD were split into quintiles following Office for National Statistics data of the English indices of deprivation 2019, with the 1st quintile being the most deprived and the 5th quintile the least deprived areas.(29) PTAL was divided into tertiles, with the 1st tertile having the worst PTAL (0, 1a, 1b) and 3rd tertile the best (5, 6a, 6b). Ethnicity was categorised as White British (White British, Irish, Any other White background), Mixed (White and Black Caribbean, White and Black African, White Asian, any other mixed background), Black (African, Caribbean, any other Black background), South Asian (Indian, Pakistani, Bangladeshi), Chinese, any other Asian background, and any other Ethnic group. Missing data points were categorised as "Unknown" group within each independent variable.

The reference category for ethnicity was the White British group, for IMD the least deprived quintile, and for PTAL the best tertile. For the rest of the independent variables, the group with the highest number of observations was considered the reference.

Patient and Public Involvement

Two patients provided insight into our discussion of the results of this study. We plan to disseminate the findings of our study to people eligible for diabetic eye screening and their families through the local press and via social media. In addition, we intend to seek wider dissemination to the public through the English national screening programme's communication team.

For peer review only - http://bmjopen.bmj.com/site/about/guidelines.xhtml

RESULTS

A total of 84,449 people were invited for a screening appointment during the study period. Mean age was 60 years (standard deviation 14.2 yrs), 53.4% were male, and 93.7% of those invited for screening had type 2 diabetes. The majority were of South Asian ethnicity (41.2%), followed by White British (29%) and Black ethnic groups (17%). 74.7% of the participants lived in areas with the highest levels of deprivation (1st and 2nd IMD quintiles). Overall, screening attendance during the study period was 83.4%.

Table 1 summarises sociodemographic characteristics of attenders and non-attenders along with crude and adjusted ORs for attendance versus non-attendance (where ORs greater than 1.0 imply greater odds of attendance).

Those aged 12 to 45 years of age showed poorer attendance when compared with the reference 46 to 60-year-old group. In adjusted analyses, participants 18 to 30 years of age were least likely to attend for screening showing a 58% reduction in the odds of attendance, and an absolute uptake difference of 18.8% when compared with the reference. After adjusting for the sociodemographic factors in table 1, the odds of attendance increase by about 17% per decade rise in age (OR= 1.17; 95%CI 1.15-1.19, p-value < 0.001).

Compared with White British individuals, those of mixed or Black ethnicity did not show any difference in the odds of attendance after adjustment. However, odds of attendance were higher amongst individuals of Asian (South Asian, Chinese and Any other Asian background) ethnicities when compared with White British individuals, even after adjustment.

Individuals living in the most deprived areas (1st IMD quintile) had a 20% reduction in the odds of attendance when compared with people living in the least deprived areas (5th IMD quintile). A linear trend suggested a 6% rise in the odds of attendance per increase in IMD quintile (p-value < 0.001).

People with longer duration of diabetes were less likely to attend. The OR per 5-year increase in duration of diabetes was 0.97 (95%CI 0.95-0.99, p-value=0.006). The average distance to screening centre was 1.7 km (IQR 1 – 2km). Only people who lived ≥ 9km from the screening centre (outside the geographical boundaries of the CCGs) were formally more likely to non-attend. Odds of attendance decreased by 1% for every km further from the screening centre, suggesting a trend (OR= 0.99; 95%CI 0.97-1.00, p-value=0.031).

Individuals with lower visual acuity (starting from visions worse than 6/9) showed a graded decline in the odds of attending the screening visit. Those with visual acuity worse than 6/18 were least likely to attend and showed a 57% reduction in odds of attendance compared with those with acuity of 6/6 to 6/9. This equates to an absolute difference in attendance of 11.4 percentage points when compared with the reference group.

For peer review only - http://bmjopen.bmj.com/site/about/guidelines.xhtml

Attendance did not appear to differ by gender, type of diabetes, or PTAL score. People registered in the screening programme for more than 5 years were more likely to attend than those registered for less than 5 years. People with >15 years of registration showed almost twice the odds of attendance than people with <5 years of registration. The OR per 5-years of registration was 1.12 (95%CI 1.08-1.17, p-value <0.001).

Table 1. Sociodemographic characteristics of attenders and non-attenders along with crude and adjusted odds ratio for attendance versus non-attendance.

For peer review only

For peer review only - http://bmjopen.bmj.com/site/about/guidelines.xhtml

Dependent: Attended*	Attended N=70405 (83.4%)	Did not attend N=14044 (16.6%)		Univariable OR (95% CI, p-value)	Multivariable Adjusted OR† (95% CI, p-value)
**AGE**					
12 – 17 years	276 (78.9)	74 (21.1)		0.73 (0.57-0.95, **p=0.016**)	0.71 (0.52-0.99, **p=0.036**)
18 – 30 years††	1003 (64.9)	543 (35.1)		0.36 (0.32-0.40, **p<0.001**)	0.42 (0.36-0.49, **p<0.001**)
31 – 45 years	8296 (77.2)	2454 (22.8)		0.66 (0.62-0.70, **p<0.001**)	0.71 (0.66-0.76, **p<0.001**)
46 – 60 years (Reference)	25779 (83.7)	5029 (16.3)		-	-
61 – 75 years	24482 (86.4)	3856 (13.6)		1.24 (1.18-1.30, **p<0.001**)	1.28 (1.21-1.35, **p<0.001**)
76 – 90 years	10109 (84.0)	1930 (16.0)		1.02 (0.97-1.08, p=0.461)	1.20 (1.11-1.29, **p<0.001**)
> 90 years††	460 (74.4)	158 (25.6)		0.57 (0.47-0.68, **p<0.001**)	0.92 (0.73-1.17, p=0.487)
**GENDER**					
Male (Reference)	37569 (83.3)	7558 (16.7)		-	-
Female	32836 (83.5)	6486 (16.5)		1.02 (0.98-1.06, p=0.323)	0.99 (0.95-1.04, p=0.717)
**ETHNICITY**					
White British (Reference)	20040 (81.9)	4435 (18.1)		-	-
Mixed	845 (77.7)	242 (22.3)		0.77 (0.67-0.90, **p=0.001**)	0.90 (0.75-1.09, p=0.264)
Black	11869 (82.9)	2454 (17.1)		1.07 (1.01-1.13, **p=0.014**)	1.02 (0.95-1.09, p=0.590)
South Asian	29708 (85.4)	5084 (14.6)		1.29 (1.24-1.35, **p<0.001**)	1.16 (1.09-1.23, **p<0.001**)
Chinese	536 (89.8)	61 (10.2)		1.94 (1.50-2.56, **p<0.001**)	1.91 (1.39-2.71, **p<0.001**)
Any other Asian background	4683 (88.0)	640 (12.0)		1.62 (1.48-1.77, **p<0.001**)	1.30 (1.17-1.45, **p<0.001**)
Any other ethnic group	2248 (83.0)	460 (17.0)		1.08 (0.97-1.20, p=0.145)	1.05 (0.92-1.20, p=0.453)
Unknown††	476 (41.6)	668 (58.4)		0.16 (0.14-0.18, **p<0.001**)	0.32 (0.27-0.38, **p<0.001**)
**IMD**					
1st quintile	20136 (81.9)	4456 (18.1)		0.77 (0.67-0.88, **p<0.001**)	0.80 (0.67-0.95, **p=0.012**)
2nd quintile	32163 (83.5)	6359 (16.5)		0.86 (0.75-0.99, **p=0.036**)	0.87 (0.73-1.03, p=0.124)
3rd quintile	12196 (84.7)	2203 (15.3)		0.94 (0.82-1.09, p=0.434)	0.94 (0.78-1.12, p=0.475)
4th quintile	4457 (85.1)	778 (14.9)		0.98 (0.84-1.14, p=0.776)	0.92 (0.75-1.11, p=0.370)
5th quintile (Reference)	1453 (85.4)	248 (14.6)		-	-
**TYPE OF DIABETES**					
Type 1 DM	2223 (75.8)	710 (24.2)		0.55 (0.51-0.60, **p<0.001**)	1.09 (0.96-1.25, p=0.190)
Type 2 DM (Reference)	67265 (85.0)	11851 (15.0)		-	-
MODY	40 (81.6)	9 (18.4)		0.78 (0.40-1.72, p=0.508)	0.85 (0.40-2.07, p=0.687)

For peer review only - http://bmjopen.bmj.com/site/about/guidelines.xhtml

Not specified/other††	877 (37.3)	1474 (62.7)		0.10 (0.10-0.11, **p<0.001**)	0.46 (0.40-0.53, **p<0.001**)
**DURATION OF DIABETES**					
1 – 10 years (Reference)	44890 (83.6)	8778 (16.4)		-	-
11 – 20 years	20327 (86.3)	3236 (13.7)		1.23 (1.18-1.28, **p<0.001**)	0.99 (0.92-1.06, p=0.727)
> 20 years	5057 (83.8)	977 (16.2)		1.01 (0.94-1.09, p=0.743)	0.87 (0.78-0.97, **p=0.011**)
Unknown††	131 (11.1)	1053 (88.9)		0.02 (0.02-0.03, **p<0.001**)	0.35 (0.26-0.47, **p<0.001**)
**DISTANCE TO CENTRE**					
≤ 1 – 2 km (Reference)	55436 (83.8)	10752 (16.2)		-	-
3 – 5 km	12895 (82.4)	2758 (17.6)		0.91 (0.87-0.95, **p<0.001**)	0.97 (0.91-1.03, p=0.301)
6 – 8 km	1044 (80.4)	254 (19.6)		0.80 (0.70-0.92, **p=0.001**)	0.90 (0.75-1.09, p=0.268)
≥ 9 km	190 (75.7)	61 (24.3)		0.60 (0.46-0.81, **p=0.001**)	0.66 (0.46-0.97, **p=0.027**)
Unknown	840 (79.3)	219 (20.7)		0.74 (0.64-0.87, **p<0.001**)	0.93 (0.77-1.12, p=0.433)
**PTAL**					
1st tertile	23281 (83.2)	4714 (16.8)		0.95 (0.90-1.01, p=0.083)	0.95 (0.89-1.02, p=0.189)
2nd tertile	36535 (83.4)	7291 (16.6)		0.96 (0.91-1.02, p=0.192)	0.97 (0.90-1.03, p=0.309)
3rd tertile (Reference)	10589 (83.9)	2039 (16.1)		-	-
**VISUAL ACUITY**					
Better than 6/6	14069 (88.7)	1798 (11.3)		0.93 (0.88-0.98, **p=0.007**)	1.08 (1.02-1.15, **p=0.007**)
6/6 to 6/9 (Reference)	52035 (89.4)	6158 (10.6)		-	-
< 6/9 to 6/18	3459 (84.7)	626 (15.3)		0.65 (0.60-0.72, **p<0.001**)	0.60 (0.55-0.66, **p<0.001**)
Worse than 6/18††	683 (78.4)	188 (21.6)		0.43 (0.37-0.51, **p<0.001**)	0.40 (0.34-0.48, **p<0.001**)
**YEARS OF REGISTRATION**					
1 – 5 years (Reference)	28809 (80.9)	6822 (19.1)		-	-
6 – 10 years	22948 (84.8)	4103 (15.2)		1.32 (1.27-1.38, **p<0.001**)	1.13 (1.07-1.20, **p<0.001**)
11 – 15 years	18242 (85.6)	3072 (14.4)		1.41 (1.34-1.47, **p<0.001**)	1.22 (1.12-1.33, **p<0.001**)
16 – 20 years	406 (89.6)	47 (10.4)		2.05 (1.53-2.81, **p<0.001**)	1.94 (1.35-2.89, **p=0.001**)

Observations are for 84,449 individuals.
Abbreviations; OR: Odds ratio, CI: Confidence Interval, PTAL: Public Transport Accessibility Level, IMD: Index of Multiple Deprivation.
* Odds ratios greater than 1 imply greater odds of attendance. † Odds ratios mutually adjusted for all factors shown in the table. †† Variable groups with uptake below the national diabetic eye screening programme uptake goal of ≥75%.
Independent variables with missing data categorised as "Unknown": type of diabetes (2.8%), duration of diabetes (1.4%), distance to screening centre (1.3%), and ethnicity (1.4%).

For peer review only

DISCUSSION

We found that people of Mixed or Black ethnicity with diabetes show very similar likelihoods of attendance at diabetic eye screening appointments compared with White British people, but that individuals of Asian race were more likely to attend than White British in this large, well organised, sociodemographically diverse urban DESP. This is the most current study with large scale data on ethnicity and diabetic eye screening. In addition, those with poorer visual acuity, younger age and residing in areas with higher levels of deprivation were less likely to attend for diabetic eye screening appointments.

Principal findings and comparison with other studies

Black, Asian and minority ethnic (BAME) groups have been reported to be more likely to develop diabetic retinopathy than White Europeans, more likely to present with sight-threatening retinopathy, (16, 37, 38) and less likely to attend for diabetic eye screening.(15, 16, 24) The commissioning and provision of diabetes eye screening programmes has improved since previous analyses were conducted. The audit was carried out in a large programme with an appointment capacity allowing re-scheduling to meet patients' availability and easily accessible by telephone and email. The cultural and language barriers perceived to prevent older people from BAME groups attending have proved to be misplaced. Attendance rates for BAME groups in our study were all higher than the White British, except for the small Mixed ethnic group, which had a lower, though non-significant, rate of attendance (4.2% uptake difference). Chinese, South Asian and any other Asian background ethnicities were most likely to attend, more so than any other ethnic group. These findings suggest that the underlying increased rates of retinopathy and sight-threatening retinopathy reported in BAME ethnic groups (38, 39) are not explained by non-attendance, raising the issue of increased susceptibility or poorer diabetic control. Although a study by Gulliford et al.(16) analysing sociodemographic inequalities in diabetic eye screening in South London had a high proportion of missing data on ethnicity (~39%), they also reported that African, Caribbean and other ethnicity groups were more likely to attend for diabetic eye screening than White Europeans. Uptake was higher amongst older people and those from BAME groups. All appointment letters are written in English, these data show that the language of the letter was no barrier to better attendance.

Socioeconomic deprivation has consistently been associated with attendance, where those from more deprived areas are less likely to attend for eye screening appointments.(15, 17, 20, 21, 26, 27) Although the overall average difference in attendance of 3.5% between most and least deprived areas found in our study is less than the 9.3% reported in earlier studies,(26) this is still greater than the 2% uptake difference found in a population from South London in 2010.(16) Our results provide further evidence of the ingrained health inequalities present in a multi-ethnic study population with high levels of deprivation. Also, we show the effect of multiple risk factors that appear to impact on attendance. Longer duration of diabetes and worsening visual acuity showed an association with non-attendance compared with individuals with shorter disease duration and better visual acuity. Previous reports have shown an association of longer duration of diabetes with non-attendance.(16, 17) Given that duration of diabetes is one of the three major risk factors for diabetic retinopathy,(4, 40, 41) and considering that >60% of people with type 2 diabetes and almost

For peer review only - http://bmjopen.bmj.com/site/about/guidelines.xhtml

all people with type 1 diabetes will have diabetic retinopathy after 20+ years duration of the disease,(40) the reduced odds of attendance observed in this group, places these patients at increased risk of visual complications. There is, to our knowledge no evidence available about the association of visual acuity with attendance to diabetic eye screening.

In other areas of the UK, increased distance from screening clinic has been associated with an increased risk of non-attendance.(20, 37) We have found that only individuals living ≥ 9 km from a screening centre were formally less likely to attend, but there was evidence of a trend in non-attendance with increasing distance. It is noteworthy that an 8 km radius from one of the NELDESP screening centre covers all of the geographic areas of the 6 CCGs, and it is possible that people living beyond 8km may have moved outside the CCG areas and not updated their GP. Interestingly, we found that the association of distance to screening centre with non-attendance is independent from PTALs in this inner-city population. This may be due to London having a well-developed public transport network and good transport-related access. These findings may not apply elsewhere, particularly to non-urban populations less served by public transport.

In accordance with previous evidence,(10, 15-17, 20, 21, 42, 43) young individuals from 12 to 45 years of age had lower odds of attendance compared with people age 46-60 years. Possible underpinning factors are over confidence about their health or demanding work schedules.(20, 25) Nonetheless, within the context of diabetes chronicity and the need for regular contact with health care services, these individuals are at increased risk of complications through longer duration of disease and possible suboptimal metabolic control.(44)

Our study has several strengths. First, a large sample size with considerable proportions of individuals from different ethnic groups representing a diverse population group all living within the programme area, with one of the most complete datasets on ethnicity reported to date. Second, the use of PTALs in addition to distance to screening centre to evidence the associations of accessibility and transport with attendance. And third, the fact that three quarters of the participants were distributed between two of the most deprived quintiles of IMD, allowing the comparative association between deprivation and ethnicity with attendance to be examined. Our study has several limitations. First, major systemic risk factors for diabetic retinopathy incidence and progression, namely hypertension and glycaemic control, were not available to include in our analysis. Second, we did not analyse the association of the sociodemographic variables with the presence of diabetic retinopathy, which although desirable, would have been difficult to ascertain for repeated non-attenders. Further work to unravel the interplay between ethnicity, deprivation and disease severity, is needed to inform strategies to improve attendance, particularly in high risk under privileged groups.

Conclusion

Smaller previous studies have reported an association between non-white ethnicities and poor attendance at diabetic eye screening appointments, however, in this large diverse urban population, South Asian, Chinese, and individuals of any other Asian background were more likely to attend for diabetic eye screening than White British people. Public health strategies

For peer review only - http://bmjopen.bmj.com/site/about/guidelines.xhtml

have in the past focussed on ethnic differences as a possible cause of variance in diabetic eye screening uptake. The data from this large cohort shows that there are other more influential factors. We have shown that worse visual acuity, higher levels of deprivation, younger age, and longer duration of diabetes are associated with non-attendance. Hence, strategies to improve uptake should be directed at these groups, in order to reduce inequalities in diabetic eye screening.

Footnotes

Contributorship statement: JA, CE, LB, AT, AR, CGO, AOB designed the study. AO-B, MS undertook data management, processing and analysis. AR, CGO, CE provided statistical advice and analysed the data. JA, CE, LB, AR, AO-B wrote the first draft of the report, which was critically appraised by all authors. All the authors approved the final draft. JA is responsible for data integrity.

Competing interests: None.

Funding: AO-B received a proportion of his funding from the Mexican National Council of Science and Technology (CONACYT). CE and AT received a proportion of their funding from the National Institute for Health Research Centre for Ophthalmology at Moorfields Eye Hospital, and UCL Institute of Ophthalmology. The views expressed in the publication are those of the authors and not necessarily those of the Department of Health.

Data sharing statement: The data that supports the findings of this study are available from the North East London Diabetic Eye Screening Programme upon reasonable request.

Patient consent for publication: Not required

Ethics approval: The study was registered as an audit and approved by the Homerton University Hospital NHS Foundation Trust Research and Innovation Department. The study adhered to the UK Data Protection Act 2018.

Transparency statement: The corresponding author affirms that the manuscript is an honest, accurate, and transparent account of the study being reported. No important aspects of the study have been omitted and all discrepancies from the study as planned have been explained.

For peer review only

References

1. Liew G, Michaelides M, Bunce C. A comparison of the causes of blindness certifications in England and Wales in working age adults (16-64 years), 1999-2000 with 2009-2010. *BMJ Open* 2014;4(2):e004015. doi: 10.1136/bmjopen-2013-004015 [published Online First: 2014/02/15]
2. Solomon SD, Chew E, Duh EJ, et al. Diabetic Retinopathy: A Position Statement by the American Diabetes Association. *Diabetes Care* 2017;40(3):412-18. doi: 10.2337/dc16-2641 [published Online First: 2017/02/23]
3. Diabetes UK. Diabetes prevalence 2019 [Available from: https://www.diabetes.org.uk/professionals/position-statements-reports/statistics/diabetes-prevalence-2019 accessed 27 Feb 2020.
4. Mathur R, Bhaskaran K, Edwards E, et al. Population trends in the 10-year incidence and prevalence of diabetic retinopathy in the UK: a cohort study in the Clinical Practice Research Datalink 2004-2014. *BMJ Open* 2017;7(2):e014444. doi: 10.1136/bmjopen-2016-014444 [published Online First: 2017/03/02]
5. Ferris FL, 3rd. How effective are treatments for diabetic retinopathy? *JAMA* 1993;269(10):1290-1. [published Online First: 1993/03/10]
6. Rohan TE, Frost CD, Wald NJ. Prevention of blindness by screening for diabetic retinopathy: a quantitative assessment. *BMJ* 1989;299(6709):1198-201. doi: 10.1136/bmj.299.6709.1198 [published Online First: 1989/11/11]
7. Scanlon PH. The English National Screening Programme for diabetic retinopathy 2003-2016. *Acta Diabetol* 2017;54(6):515-25. doi: 10.1007/s00592-017-0974-1 [published Online First: 2017/02/23]
8. Public Health England. Diabetic eye screening standards valid for data collected from 1 April 2019 2019 [Available from: https://www.gov.uk/government/publications/diabetic-eye-screening-programme-standards/diabetic-eye-screening-standards-valid-for-data-collected-from-1-april-2019#des-s07-uptake-routine-digital-screening accessed 27 Feb 2020.
9. Public Health England. Diabetic Eye Screening: 2016 to 2017 data 2017 [Available from: https://www.gov.uk/government/publications/diabetic-eye-screening-2016-to-2017-data accessed 01, July 2020.
10. Forster AS, Forbes A, Dodhia H, et al. Non-attendance at diabetic eye screening and risk of sight-threatening diabetic retinopathy: a population-based cohort study. *Diabetologia* 2013;56(10):2187-93. doi: 10.1007/s00125-013-2975-0 [published Online First: 2013/06/25]
11. Virk R, Binns AM, Chambers R, et al. How is the risk of being diagnosed with referable diabetic retinopathy affected by failure to attend diabetes eye screening appointments? *Eye (Lond)* 2020 doi: 10.1038/s41433-020-0877-1 [published Online First: 2020/04/23]
12. Borrell C, Pons-Vigues M, Morrison J, et al. Factors and processes influencing health inequalities in urban areas. *J Epidemiol Community Health* 2013;67(5):389-91. doi: 10.1136/jech-2012-202014 [published Online First: 2013/02/16]
13. Riva M, Curtis S, Gauvin L, et al. Unravelling the extent of inequalities in health across urban and rural areas: evidence from a national sample in England. *Soc Sci Med* 2009;68(4):654-63. doi: 10.1016/j.socscimed.2008.11.024 [published Online First: 2008/12/26]

For peer review only - http://bmjopen.bmj.com/site/about/guidelines.xhtml

14. Dorling D. Editorial—Inequality and Injustice: Some News from Britain. *Urban Geography* 2012;33(5):621-29. doi: 10.2747/0272-3638.33.5.621
15. Millett C, Dodhia H. Diabetes retinopathy screening: audit of equity in participation and selected outcomes in South East London. *J Med Screen* 2006;13(3):152-5. doi: 10.1258/096914106778440608 [published Online First: 2006/09/30]
16. Gulliford MC, Dodhia H, Chamley M, et al. Socio-economic and ethnic inequalities in diabetes retinal screening. *Diabet Med* 2010;27(3):282-8. doi: 10.1111/j.1464-5491.2010.02946.x [published Online First: 2010/06/12]
17. Leese GP, Boyle P, Feng Z, et al. Screening uptake in a well-established diabetic retinopathy screening program: the role of geographical access and deprivation. *Diabetes Care* 2008;31(11):2131-5. doi: 10.2337/dc08-1098 [published Online First: 2008/08/30]
18. Dervan E, Lillis D, Flynn L, et al. Factors that influence the patient uptake of diabetic retinopathy screening. *Ir J Med Sci* 2008;177(4):303-8. doi: 10.1007/s11845-008-0192-5 [published Online First: 2008/07/22]
19. van Eijk KN, Blom JW, Gussekloo J, et al. Diabetic retinopathy screening in patients with diabetes mellitus in primary care: Incentives and barriers to screening attendance. *Diabetes Res Clin Pract* 2012;96(1):10-6. doi: 10.1016/j.diabres.2011.11.003 [published Online First: 2011/12/06]
20. Moreton RBR, Stratton IM, Chave SJ, et al. Factors determining uptake of diabetic retinopathy screening in Oxfordshire. *Diabet Med* 2017;34(7):993-99. doi: 10.1111/dme.13350 [published Online First: 2017/03/16]
21. Orton E, Forbes-Haley A, Tunbridge L, et al. Equity of uptake of a diabetic retinopathy screening programme in a geographically and socio-economically diverse population. *Public Health* 2013;127(9):814-21. doi: 10.1016/j.puhe.2013.04.015 [published Online First: 2013/08/27]
22. Gange WS, Xu BY, Lung K, et al. Rates of Eye Care and Diabetic Eye Disease Among Insured Patients with Newly-diagnosed Type 2 Diabetes. *Ophthalmol Retina* 2020 doi: 10.1016/j.oret.2020.07.004 [published Online First: 2020/07/13]
23. Al-Alawi A, Al-Hassan A, Chauhan D, et al. Knowledge, Attitude, and Perception of Barriers for Eye Care among Diabetic Persons Registered at Employee Health Department of a Tertiary Eye Hospital of Central Saudi Arabia. *Middle East Afr J Ophthalmol* 2016;23(1):71-4. doi: 10.4103/0974-9233.164629 [published Online First: 2016/03/10]
24. Fathy C, Patel S, Sternberg P, Jr., et al. Disparities in Adherence to Screening Guidelines for Diabetic Retinopathy in the United States: A Comprehensive Review and Guide for Future Directions. *Semin Ophthalmol* 2016;31(4):364-77. doi: 10.3109/08820538.2016.1154170 [published Online First: 2016/04/27]
25. Lindenmeyer A, Sturt JA, Hipwell A, et al. Influence of primary care practices on patients' uptake of diabetic retinopathy screening: a qualitative case study. *Br J Gen Pract* 2014;64(625):e484-92. doi: 10.3399/bjgp14X680965 [published Online First: 2014/07/30]
26. Scanlon PH, Carter SC, Foy C, et al. Diabetic retinopathy and socioeconomic deprivation in Gloucestershire. *J Med Screen* 2008;15(3):118-21. doi: 10.1258/jms.2008.008013 [published Online First: 2008/10/18]
27. Waqar S, Bullen G, Chant S, et al. Cost implications, deprivation and geodemographic segmentation analysis of non-attenders (DNA) in an established diabetic retinopathy

For peer review only - http://bmjopen.bmj.com/site/about/guidelines.xhtml

screening programme. *Diabetes Metab Syndr* 2012;6(4):199-202. doi: 10.1016/j.dsx.2012.08.009 [published Online First: 2012/12/04]
28. Public Health England. Screening Quality Assurance Visit Report. NHS Diabetic Eye Screening Service North East London Diabetic Eye Screening Programme. 2017
29. Ministry of Housing Communities & Local Government. The English Indices of Deprivation 2019 (IoD 2019). 2019
30. Pittini M, Rocks C, Leeser R. London geographies and economic performance – insights from the Local Industrial Strategy evidence base interim report. *GLA Economics* 2019(215):12-16.
31. Office of National Statistics. Ethnicity and National Identity in England and Wales: 2011 2011 [Available from: https://www.ons.gov.uk/peoplepopulationandcommunity/culturalidentity/ethnicity/articles/ethnicityandnationalidentityinenglandandwales/2012-12-11#measuring-ethnicity accessed April 13th 2020.
32. Government UK. UK population by ethnicity. Regional Ethnic Diversity 2011 [Available from: https://www.ethnicity-facts-figures.service.gov.uk/uk-population-by-ethnicity/national-and-regional-populations/regional-ethnic-diversity/latest accessed 5 July 2020.
33. Olvera-Barrios A, Heeren TF, Balaskas K, et al. Comparison of true-colour wide-field confocal scanner imaging with standard fundus photography for diabetic retinopathy screening. *Br J Ophthalmol* 2020 doi: 10.1136/bjophthalmol-2019-315269 [published Online First: 2020/03/07]
34. Transport for London. Public Transport Accessibility Levels 2017 [Available from: https://data.london.gov.uk/dataset/public-transport-accessibility-levels#:~:text=The%20method%20is%20essentially%20a,excellent%20access%20to%20public%20transport. accessed 6 July 2020.
35. Transport for London. WebCAT planning tool 2017 [Available from: https://tfl.gov.uk/info-for/urban-planning-and-construction/planning-with-webcat/webcat?intcmp=25932 accessed 6 July 2020.
36. R: A Language and Environment for Statistical Computing [program]. Viena, Austria: R Foundation for Statistical Computing, 2020.
37. Kliner M, Fell G, Gibbons C, et al. Diabetic retinopathy equity profile in a multi-ethnic, deprived population in Northern England. *Eye (Lond)* 2012;26(5):671-7. doi: 10.1038/eye.2012.3 [published Online First: 2012/02/04]
38. Sivaprasad S, Gupta B, Gulliford MC, et al. Ethnic variation in the prevalence of visual impairment in people attending diabetic retinopathy screening in the United Kingdom (DRIVE UK). *PLoS One* 2012;7(6):e39608. doi: 10.1371/journal.pone.0039608 [published Online First: 2012/07/05]
39. Sivaprasad S, Gupta B, Crosby-Nwaobi R, et al. Prevalence of diabetic retinopathy in various ethnic groups: a worldwide perspective. *Surv Ophthalmol* 2012;57(4):347-70. doi: 10.1016/j.survophthal.2012.01.004 [published Online First: 2012/05/01]
40. Fong DS, Aiello L, Gardner TW, et al. Retinopathy in diabetes. *Diabetes Care* 2004;27 Suppl 1:S84-7. doi: 10.2337/diacare.27.2007.s84 [published Online First: 2003/12/25]
41. Yau JW, Rogers SL, Kawasaki R, et al. Global prevalence and major risk factors of diabetic retinopathy. *Diabetes Care* 2012;35(3):556-64. doi: 10.2337/dc11-1909 [published Online First: 2012/02/04]

For peer review only - http://bmjopen.bmj.com/site/about/guidelines.xhtml

42. Scanlon PH, Stratton IM, Leese GP, et al. Screening attendance, age group and diabetic retinopathy level at first screen. *Diabet Med* 2016;33(7):904-11. doi: 10.1111/dme.12957 [published Online First: 2015/09/05]

43. Ahmad B, Neilson F. A Health Equity Audit of the Diabetic Eye Screening Programmes in Cumbria and the North East. *British Journal of Diabetes* 2019;19(1):42-44. doi: 10.15277/bjd.2019.203

44. Hainsworth DP, Bebu I, Aiello LP, et al. Risk Factors for Retinopathy in Type 1 Diabetes: The DCCT/EDIC Study. *Diabetes Care* 2019;42(5):875-82. doi: 10.2337/dc18-2308 [published Online First: 2019/03/06]

For peer review only

STROBE Statement—Checklist of items that should be included in reports of ***cohort studies***

	Item No	Recommendation	Page No
**Title and abstract**	1	(*a*) Indicate the study's design with a commonly used term in the title or the abstract	1-2
		(*b*) Provide in the abstract an informative and balanced summary of what was done and what was found	2
**Introduction**			
Background/rationale	2	Explain the scientific background and rationale for the investigation being reported	4
Objectives	3	State specific objectives, including any prespecified hypotheses	4
**Methods**			
Study design	4	Present key elements of study design early in the paper	5
Setting	5	Describe the setting, locations, and relevant dates, including periods of recruitment, exposure, follow-up, and data collection	5
Participants	6	(*a*) Give the eligibility criteria, and the sources and methods of selection of participants. Describe methods of follow-up	5
		(*b*) For matched studies, give matching criteria and number of exposed and unexposed	NA
Variables	7	Clearly define all outcomes, exposures, predictors, potential confounders, and effect modifiers. Give diagnostic criteria, if applicable	5-6
Data sources/ measurement	8*	For each variable of interest, give sources of data and details of methods of assessment (measurement). Describe comparability of assessment methods if there is more than one group	5-6
Bias	9	Describe any efforts to address potential sources of bias	5-6
Study size	10	Explain how the study size was arrived at	5-6
Quantitative variables	11	Explain how quantitative variables were handled in the analyses. If applicable, describe which groupings were chosen and why	5-6
Statistical methods	12	(*a*) Describe all statistical methods, including those used to control for confounding	6
		(*b*) Describe any methods used to examine subgroups and interactions	6
		(*c*) Explain how missing data were addressed	6
		(*d*) If applicable, explain how loss to follow-up was addressed	NA
		(*e*) Describe any sensitivity analyses	NA
**Results**			
Participants	13*	(a) Report numbers of individuals at each stage of study—eg numbers potentially eligible, examined for eligibility, confirmed eligible, included in the study, completing follow-up, and analysed	7
		(b) Give reasons for non-participation at each stage	7
		(c) Consider use of a flow diagram	7
Descriptive data	14*	(a) Give characteristics of study participants (eg demographic, clinical, social) and information on exposures and potential confounders	7
		(b) Indicate number of participants with missing data for each variable of interest	10
		(c) Summarise follow-up time (eg, average and total amount)	NA
Outcome data	15*	Report numbers of outcome events or summary measures over time	7-10

For peer review only

For peer review only - http://bmjopen.bmj.com/site/about/guidelines.xhtml

Main results	16	(*a*) Give unadjusted estimates and, if applicable, confounder-adjusted estimates and their precision (eg, 95% confidence interval). Make clear which confounders were adjusted for and why they were included	7-10
		(*b*) Report category boundaries when continuous variables were categorized	7-10
		(*c*) If relevant, consider translating estimates of relative risk into absolute risk for a meaningful time period	NA
Other analyses	17	Report other analyses done—eg analyses of subgroups and interactions, and sensitivity analyses	7
**Discussion**			
Key results	18	Summarise key results with reference to study objectives	11-12
Limitations	19	Discuss limitations of the study, taking into account sources of potential bias or imprecision. Discuss both direction and magnitude of any potential bias	12
Interpretation	20	Give a cautious overall interpretation of results considering objectives, limitations, multiplicity of analyses, results from similar studies, and other relevant evidence	11-12
Generalisability	21	Discuss the generalisability (external validity) of the study results	11-12
**Other information**			
Funding	22	Give the source of funding and the role of the funders for the present study and, if applicable, for the original study on which the present article is based	13

*Give information separately for exposed and unexposed groups.

Note: An Explanation and Elaboration article discusses each checklist item and gives methodological background and published examples of transparent reporting. The STROBE checklist is best used in conjunction with this article (freely available on the Web sites of PLoS Medicine at http://www.plosmedicine.org/, Annals of Internal Medicine at http://www.annals.org/, and Epidemiology at http://www.epidem.com/). Information on the STROBE Initiative is available at http://www.strobe-statement.org.

For peer review only

The effect of ethnicity and other sociodemographic factors on attendance at diabetic eye screening: a 12-month retrospective cohort study

Journal:	*BMJ Open*
Manuscript ID	bmjopen-2020-046264.R1
Article Type:	Original research
Date Submitted by the Author:	10-May-2021
Complete List of Authors:	Olvera-Barrios, Abraham; Moorfields Eye Hospital NHS Foundation Trust, Medical Retina; University College London Institute of Ophthalmology<br>Seltene, Michael; Moorfields Eye Hospital NHS Foundation Trust, Diabetic Retinal Screening<br>Heeren, Tjebo; Moorfields Eye Hospital NHS Foundation Trust, Medical Retina; University College London Institute of Ophthalmology<br>Chambers, Ryan; Homerton University Hospital NHS Foundation Trust<br>Bolter, Louis; Homerton University Hospital NHS Foundation Trust<br>Tufail, Adnan; Moorfields Eye Hospital NHS Foundation Trust, ; UCL Institute of Ophthmology,<br>Owen, C; St George's, University London, Population Health Research Institute<br>Rudnicka, Alicja; St George's University of London, Population Health Research Institute<br>Egan, Catherine; Moorfields Eye Hospital; University College London Institute of Ophthalmology<br>Anderson, John; Homerton University Hospital NHS Foundation Trust
**Primary Subject Heading**:	Ophthalmology
Secondary Subject Heading:	Epidemiology, Diabetes and endocrinology, Public health
Keywords:	Diabetic retinopathy < DIABETES & ENDOCRINOLOGY, EPIDEMIOLOGY, SOCIAL MEDICINE, Clinical audit < HEALTH SERVICES ADMINISTRATION & MANAGEMENT, Quality in health care < HEALTH SERVICES ADMINISTRATION & MANAGEMENT

SCHOLARONE™ Manuscripts

I, the Submitting Author has the right to grant and does grant on behalf of all authors of the Work (as defined in the below author licence), an exclusive licence and/or a non-exclusive licence for contributions from authors who are: i) UK Crown employees; ii) where BMJ has agreed a CC-BY licence shall apply, and/or iii) in accordance with the terms applicable for US Federal Government officers or employees acting as part of their official duties; on a worldwide, perpetual, irrevocable, royalty-free basis to BMJ Publishing Group Ltd ("BMJ") its licensees and where the relevant Journal is co-owned by BMJ to the co-owners of the Journal, to publish the Work in this journal and any other BMJ products and to exploit all rights, as set out in our licence.

The Submitting Author accepts and understands that any supply made under these terms is made by BMJ to the Submitting Author unless you are acting as an employee on behalf of your employer or a postgraduate student of an affiliated institution which is paying any applicable article publishing charge ("APC") for Open Access articles. Where the Submitting Author wishes to make the Work available on an Open Access basis (and intends to pay the relevant APC), the terms of reuse of such Open Access shall be governed by a Creative Commons licence – details of these licences and which Creative Commons licence will apply to this Work are set out in our licence referred to above.

Other than as permitted in any relevant BMJ Author's Self Archiving Policies, I confirm this Work has not been accepted for publication elsewhere, is not being considered for publication elsewhere and does not duplicate material already published. I confirm all authors consent to publication of this Work and authorise the granting of this licence.

For peer review only

The effect of ethnicity and other sociodemographic factors on attendance at diabetic eye screening: a 12-month retrospective cohort study

Abraham Olvera-Barrios,[1-2] Michael Seltene,[3] Tjebo F.C. Heeren,[1-2] Ryan Chambers,[4] Louis Bolter,[4] Adnan Tufail,[1-2] Christopher G. Owen,[5] Alicja R. Rudnicka,[5] Catherine A. Egan,[1-2] John Anderson.[4]

1. Medical Retina, Moorfields Eye Hospital NHS Foundation Trust, London, United Kingdom
2. University College London Institute of Ophthalmology, London, United Kingdom
3. Diabetic Retinopathy Screening, Moorfields Eye Hospital NHS Foundation Trust, London, United Kingdom
4. Diabetes, Homerton University Hospital NHS Foundation Trust, London, United Kingdom
5. Population Health Research Institute, St George's University of London, London, United Kingdom

Corresponding Author
John Anderson
Homerton University Hospital NHS Foundation Trust
john.anderson@nhs.net

Word count: 2814

For peer review only - http://bmjopen.bmj.com/site/about/guidelines.xhtml

ABSTRACT

Objectives: To examine the association of sociodemographic characteristics with attendance at diabetic eye screening in a large ethnically diverse urban population.

Design: Retrospective cohort study.

Setting: Screening visits in the North East London Diabetic Eye Screening Programme (NELDESP).

Participants: 84,449 people with diabetes aged 12 years or older registered in the NELDESP and scheduled for screening between 1st April 2017 to 31st March 2018.

Main outcome measure: Association between sociodemographic factors (age, gender, self-defined ethnicity, area level deprivation), type of diabetes, duration of diabetes, visual acuity, years of NELDESP registration, distance to screening centre, and Public Transport Accessibility, with attendance for diabetic eye screening.

Results: The mean age of people with diabetes was 60 yrs (SD 14.2 yrs), 53.4% were males, 41% South Asian, 29% White British and 17% Black; 83.4 % attended screening. Black people with diabetes had similar levels of attendance compared with White British people. However, South Asian, Chinese and any other Asian background ethnicities showed greater odds of attendance compared with White British. When compared with their respective reference group, high levels of deprivation, younger age, longer duration of diabetes, worse visual acuity and longer distance to screening centre, were all associated with non-attendance. There was a higher likelihood of attendance per quintile improvement in deprivation (odds ratio [OR], 1.06; 95%CI, 1.03-1.08), with increasing age (OR per decade, 1.17; 95%CI, 1.15-1.19), with better visual acuity (OR per Bailey-Lovey chart line 1.12, 95%CI 1.11-1.14) and with longer time of NELDESP registration (OR per 5 yrs, 1.12; 95%CI, 1.08-1.17).

Conclusion: Ethnic differences in diabetic eye screening uptake are evident, but despite preconceptions a higher likelihood of screening attendance was observed among Asians compared with whites. Poorer socioeconomic profile was associated with higher likelihood of non-attendance for screening. Further work is needed to understand how to target individuals at risk of non-attendance and reduce inequalities.

For peer review only - http://bmjopen.bmj.com/site/about/guidelines.xhtml

Article summary

Strengths and Limitations of the study

- We used a retrospective cohort of 84,449 people to address a key issue in diabetic retinopathy screening: The association of sociodemographic factors with non-attendance to a systematic diabetic eye screening programme.
- Strengths contain that our study is one of the most current analysis with high-quality data on ethnicity, a diverse population with high socioeconomic deprivation, and the inclusion of additional factors, such as, the distance to screening centre and public transport accessibility level.
- Systemic risk factors for diabetic retinopathy incidence and progression, and the association of sociodemographic variables with diabetic retinopathy were not available to analyse.
- Our study cohort is from people with health coverage registered in a systematic diabetic eye screening programme, hence results cannot be extrapolated to populations from different settings.

For peer review only

For peer review only - http://bmjopen.bmj.com/site/about/guidelines.xhtml

INTRODUCTION

Diabetic retinopathy is a common neurovascular complication of diabetes and a major cause of blindness.(1, 2) There are at least 3.9 million people diagnosed with diabetes in the United Kingdom, a number expected to rise to 5.8 million by 2025.(3) It is estimated that 30% of people with diabetes will develop retinopathy, and about 9% will develop sight-threatening retinopathy.(4) An early diagnosis through population screening, timely referral and treatment are essential for prevention of diabetes-related visual impairment.(5-7) The UK implemented the first systematically organised diabetic eye screening programme (DESP) in the world in England in 2003, achieving nation-wide coverage by 2008. The English DESP offers annual mydriatic photographic screening to all people with diabetes aged 12 or older.(7) In accordance with national standards, screening of ≥85% of the eligible diabetic population is considered achievable, however, English DESP uptake data from 2016-2017 showed that this was not met in 75% of London's Clinical Commissioning Groups (CCGs) areas.(8, 9) Regional differences in screening delivery and uptake may explain regional variation in diabetic eye disease.(4)

Non-attendance at annual diabetic eye screening visits has been associated with late presentation of sight-threatening retinopathy.(10, 11) Inequalities in health tend to be present in urban areas with contrasting sociodemographic conditions. London, a metropolis where people from the extremes of the deprivation indices live side-by-side, is a remarkable example of how these inequalities can result in different uptake rates across and within boroughs.(12-14) Health inequalities can create significant attendance variation among subgroups, and are of concern to any screening programme. Sociodemographic factors such as, age (15-22), gender,(22-24) ethnicity,(15, 16, 24) transportation,(25) and socioeconomic deprivation (15-17, 20-22, 24, 26, 27) have all been associated with non-attendance.

The North East London population is sociodemographically diverse, with a wide variation in ethnicities and a varied health profile with higher than average level of deprivation and a lower than average life expectancy.(28-30) The North East London DESP (NELDESP) serves a total eligible population of approximately 125 000 people with diabetes aged 12 and over.(28) The NELDESP aims to invite ≥98% of eligible individuals and to have an uptake ≥85%. We examined the sociodemographic determinants of attendance at the NELDESP, within this multi-ethnic population with high levels of deprivation.

For peer review only - http://bmjopen.bmj.com/site/about/guidelines.xhtml

METHODS

We performed a 12-month retrospective cohort study between 1st April 2017 to 31st March 2018. The study was registered and approved as an audit through the research governance process at the Homerton University Hospital NHS Foundation Trust.

Setting

The NELDESP is provided by the Homerton University Hospital NHS Foundation Trust. We analysed data from people with diabetes living in 6 CCG areas with inner city multi-ethnic populations, residing in London boroughs of Newham, Redbridge, Tower Hamlets and Waltham Forest, which have been classified as the most ethnically diverse in London;(31, 32) Barking & Dagenham and Hackney, which have a substantial multi-ethnic population.

The NELDESP is run according to English DESP guidelines. All people with diabetes aged 12 or older are identified through coding in primary care electronic record systems. Software is used to generate invitations to attend for NELDESP appointments. The Homerton Hospital carries out appointment call/recall, screening, image grading, referral tasks, and is responsible for providing clinical leadership and programme management, including failsafe procedures and internal quality assurance.(28)

Briefly, a screening visit entails a visual acuity assessment, and pupil dilation to obtain two 45° digital retinal images of each eye, centred on the fovea and disc, respectively. We have described in detail the imaging, grading protocol and referral pathway elsewhere.(33)

Data extraction

We carried out an anonymised data extraction of all screening appointments between the study period using structure query language (SQL) searches. An anonymised data base for analysis was created.

Independent variable recording

Ethnicity

Self-classified ethnicity data was collected from patients at the time of screening, or from the routinely recorded ethnicity data provided by their GP surgery. Their ethnicity was recorded in the nationally mandated screening software in accordance with the 2011 Office for National Statistics census groups.(31)

Index of multiple deprivation (IMD)

The English indices of deprivation are composed of 39 indicators arranged in 7 different domains of deprivation, which are combined and weighted to create the IMD, the official measure of relative deprivation in England. This measure is calculated for every neighbourhood or small area (lower-layer super output area [LSOA]) in England. There are 32,844 LSOAs with an average population of 1,500, and each of them is ranked from 1st, the most deprived area, to 32, the 844th

For peer review only - http://bmjopen.bmj.com/site/about/guidelines.xhtml

least deprived area. Patient's postcodes were linked to their LSOA indices of multiple deprivation scores.

Visual acuity, distance and Public Transport Accessibility Level (PTAL)

We recorded the most recent visual acuity within a 3-year time frame in Snellen notation for the analysis. The better-seeing eye visual acuity score was assigned to each person. We calculated distance to screening centre (in kilometres) as a straight line from the patient's postcode to the destination. The PTAL is a metric tool from Transport for London which rates locations by distance to the public transport network, thus reflecting the accessibility to public transport within Greater London. The PTAL grade takes into account walk access time, average waiting time, service availability and service reliability. The grading has 9 levels from 0 (with the poorest access) to 6b (excellent access).(34) Using Transport for London's Web-based Connectivity Assessment Toolkit (WebCAT),(35) we extracted the PTALs for each patient's home postcode.

Statistical analysis

We used R version 4.0.0 for statistical analysis.(36) We conducted a multivariable logistic regression analysis of attendance at screening visit (binary outcome coded "1" if patient attended and "0" if they did not attend). A test for trend was performed if the odds ratios showed a linear pattern across categorical variables. Attendance was defined as a participant completing the diabetic retinopathy screening process. Independent variables considered were age, gender, ethnicity, IMD, type of diabetes, duration of diabetes, visual acuity, years of registration into the DESP, distance to screening centre, and PTAL.

We categorised continuous variables for the analysis to allow for non-linear patterns in attendance. Rank scores of the IMD were split into quintiles following Office for National Statistics data of the English indices of deprivation 2019, with the 1st quintile being the most deprived and the 5th quintile the least deprived areas.(29) PTAL was divided into tertiles, with the 1st tertile having the worst PTAL (0, 1a, 1b) and 3rd tertile the best (5, 6a, 6b). Ethnicity was categorised as White British (White British, Irish, Any other White background), Mixed (White and Black Caribbean, White and Black African, White Asian, any other mixed background), Black (African, Caribbean, any other Black background), South Asian (Indian, Pakistani, Bangladeshi), Chinese, any other Asian background, and any other Ethnic group. Missing data points were categorised as "Unknown" group within each independent variable.

The reference category for ethnicity was the White British group, for IMD the least deprived quintile, and for PTAL the best tertile. For the rest of the independent variables, the group with the highest number of observations was considered the reference.

Patient and Public Involvement

Two patients provided insight into our discussion of the results of this study. We plan to disseminate the findings of our study to people eligible for diabetic eye screening and their families through the local press and via social media. In addition, we intend to seek wider dissemination to the public through the English national screening programme's communication team.

For peer review only - http://bmjopen.bmj.com/site/about/guidelines.xhtml

RESULTS

A total of 84,449 people were invited for a screening appointment during the study period. Mean age was 60 years (standard deviation 14.2 yrs), 53.4% were male, and 93.7% of those invited for screening had type 2 diabetes. The majority were of South Asian ethnicity (41.2%), followed by White British (29%) and Black ethnic groups (17%). 74.7% of the participants lived in areas with the highest levels of deprivation (1st and 2nd IMD quintiles). Overall, screening attendance during the study period was 83.4%.

Table 1 summarises sociodemographic characteristics of attenders and non-attenders along with crude and adjusted ORs for attendance versus non-attendance (where ORs greater than 1.0 imply greater odds of attendance).

Those aged 12 to 45 years of age showed poorer attendance when compared with the reference 46 to 60-year-old group. In adjusted analyses, participants 18 to 30 years of age were least likely to attend for screening showing a 58% reduction in the odds of attendance, and an absolute uptake difference of 18.8% when compared with the reference. After adjusting for the sociodemographic factors in table 1, the odds of attendance increase by about 17% per decade rise in age (OR= 1.17; 95%CI 1.15-1.19, p-value < 0.001).

Compared with White British individuals, those of mixed or Black ethnicity did not show any difference in the odds of attendance after adjustment. However, odds of attendance were higher amongst individuals of Asian (South Asian, Chinese and Any other Asian background) ethnicities when compared with White British individuals, even after adjustment.

Individuals living in the most deprived areas (1st IMD quintile) had a 20% reduction in the odds of attendance when compared with people living in the least deprived areas (5th IMD quintile). A linear trend suggested a 6% rise in the odds of attendance per increase in IMD quintile (p-value < 0.001).

People with longer duration of diabetes were less likely to attend. The OR per 5-year increase in duration of diabetes was 0.97 (95%CI 0.95-0.99, p-value=0.006). The average distance to screening centre was 1.7 km (IQR 1 – 2km). Only people who lived ≥ 9km from the screening centre (outside the geographical boundaries of the CCGs) were formally more likely to non-attend. Odds of attendance decreased by 1% for every km further from the screening centre, suggesting a trend (OR= 0.99; 95%CI 0.97-1.00, p-value=0.031).

Individuals with lower visual acuity (starting from visions worse than 6/9) showed a graded decline in the odds of attending the screening visit. Those with visual acuity worse than 6/18 were least likely to attend and showed a 57% reduction in odds of attendance compared with those with acuity of 6/6 to 6/9. This equates to an absolute difference in attendance of 11.4 percentage points when compared with the reference group.

For peer review only - http://bmjopen.bmj.com/site/about/guidelines.xhtml

Attendance did not appear to differ by gender, type of diabetes, or PTAL score. People registered in the screening programme for more than 5 years were more likely to attend than those registered for less than 5 years. People with >15 years of registration showed almost twice the odds of attendance than people with <5 years of registration. The OR per 5-years of registration was 1.12 (95%CI 1.08-1.17, p-value <0.001).

For peer review only

Table 1. Sociodemographic characteristics of attenders and non-attenders along with crude and adjusted odds ratio for attendance versus non-attendance.

For peer review only - http://bmjopen.bmj.com/site/about/guidelines.xhtml

Dependent: Attended*	Attended N=70405 (83.4%)	Did not attend N=14044 (16.6%)		Univariable OR (95% CI, p-value)	Multivariable Adjusted OR† (95% CI, p-value)
**AGE**					
12 – 17 years	276 (78.9)	74 (21.1)		0.73 (0.57-0.95, **p=0.016**)	0.71 (0.52-0.99, **p=0.036**)
18 – 30 years††	1003 (64.9)	543 (35.1)		0.36 (0.32-0.40, **p<0.001**)	0.42 (0.36-0.49, **p<0.001**)
31 – 45 years	8296 (77.2)	2454 (22.8)		0.66 (0.62-0.70, **p<0.001**)	0.71 (0.66-0.76, **p<0.001**)
46 – 60 years (Reference)	25779 (83.7)	5029 (16.3)		-	-
61 – 75 years	24482 (86.4)	3856 (13.6)		1.24 (1.18-1.30, **p<0.001**)	1.28 (1.21-1.35, **p<0.001**)
76 – 90 years	10109 (84.0)	1930 (16.0)		1.02 (0.97-1.08, p=0.461)	1.20 (1.11-1.29, **p<0.001**)
> 90 years††	460 (74.4)	158 (25.6)		0.57 (0.47-0.68, **p<0.001**)	0.92 (0.73-1.17, p=0.487)
**GENDER**					
Male (Reference)	37569 (83.3)	7558 (16.7)		-	-
Female	32836 (83.5)	6486 (16.5)		1.02 (0.98-1.06, p=0.323)	0.99 (0.95-1.04, p=0.717)
**ETHNICITY**					
White British (Reference)	20040 (81.9)	4435 (18.1)		-	-
Mixed	845 (77.7)	242 (22.3)		0.77 (0.67-0.90, **p=0.001**)	0.90 (0.75-1.09, p=0.264)
Black	11869 (82.9)	2454 (17.1)		1.07 (1.01-1.13, **p=0.014**)	1.02 (0.95-1.09, p=0.590)
South Asian	29708 (85.4)	5084 (14.6)		1.29 (1.24-1.35, **p<0.001**)	1.16 (1.09-1.23, **p<0.001**)
Chinese	536 (89.8)	61 (10.2)		1.94 (1.50-2.56, **p<0.001**)	1.91 (1.39-2.71, **p<0.001**)
Any other Asian background	4683 (88.0)	640 (12.0)		1.62 (1.48-1.77, **p<0.001**)	1.30 (1.17-1.45, **p<0.001**)
Any other ethnic group	2248 (83.0)	460 (17.0)		1.08 (0.97-1.20, p=0.145)	1.05 (0.92-1.20, p=0.453)
Unknown††	476 (41.6)	668 (58.4)		0.16 (0.14-0.18, **p<0.001**)	0.32 (0.27-0.38, **p<0.001**)
**IMD**					
1st quintile	20136 (81.9)	4456 (18.1)		0.77 (0.67-0.88, **p<0.001**)	0.80 (0.67-0.95, **p=0.012**)
2nd quintile	32163 (83.5)	6359 (16.5)		0.86 (0.75-0.99, **p=0.036**)	0.87 (0.73-1.03, p=0.124)
3rd quintile	12196 (84.7)	2203 (15.3)		0.94 (0.82-1.09, p=0.434)	0.94 (0.78-1.12, p=0.475)
4th quintile	4457 (85.1)	778 (14.9)		0.98 (0.84-1.14, p=0.776)	0.92 (0.75-1.11, p=0.370)
5th quintile (Reference)	1453 (85.4)	248 (14.6)		-	-
**TYPE OF DIABETES**					
Type 1 DM	2223 (75.8)	710 (24.2)		0.55 (0.51-0.60, **p<0.001**)	1.09 (0.96-1.25, p=0.190)
Type 2 DM (Reference)	67265 (85.0)	11851 (15.0)		-	-
MODY	40 (81.6)	9 (18.4)		0.78 (0.40-1.72, p=0.508)	0.85 (0.40-2.07, p=0.687)

For peer review only - http://bmjopen.bmj.com/site/about/guidelines.xhtml

Not specified/other††	877 (37.3)	1474 (62.7)		0.10 (0.10-0.11, **p<0.001**)	0.46 (0.40-0.53, **p<0.001**)
**DURATION OF DIABETES**					
1 – 10 years (Reference)	44890 (83.6)	8778 (16.4)		-	-
11 – 20 years	20327 (86.3)	3236 (13.7)		1.23 (1.18-1.28, **p<0.001**)	0.99 (0.92-1.06, p=0.727)
> 20 years	5057 (83.8)	977 (16.2)		1.01 (0.94-1.09, p=0.743)	0.87 (0.78-0.97, **p=0.011**)
Unknown††	131 (11.1)	1053 (88.9)		0.02 (0.02-0.03, **p<0.001**)	0.35 (0.26-0.47, **p<0.001**)
**DISTANCE TO CENTRE**					
≤ 1 – 2 km (Reference)	55436 (83.8)	10752 (16.2)		-	-
3 – 5 km	12895 (82.4)	2758 (17.6)		0.91 (0.87-0.95, **p<0.001**)	0.97 (0.91-1.03, p=0.301)
6 – 8 km	1044 (80.4)	254 (19.6)		0.80 (0.70-0.92, **p=0.001**)	0.90 (0.75-1.09, p=0.268)
≥ 9 km	190 (75.7)	61 (24.3)		0.60 (0.46-0.81, **p=0.001**)	0.66 (0.46-0.97, **p=0.027**)
Unknown	840 (79.3)	219 (20.7)		0.74 (0.64-0.87, **p<0.001**)	0.93 (0.77-1.12, p=0.433)
**PTAL**					
1st tertile	23281 (83.2)	4714 (16.8)		0.95 (0.90-1.01, p=0.083)	0.95 (0.89-1.02, p=0.189)
2nd tertile	36535 (83.4)	7291 (16.6)		0.96 (0.91-1.02, p=0.192)	0.97 (0.90-1.03, p=0.309)
3rd tertile (Reference)	10589 (83.9)	2039 (16.1)		-	-
**VISUAL ACUITY**					
Better than 6/6	14069 (88.7)	1798 (11.3)		0.93 (0.88-0.98, **p=0.007**)	1.08 (1.02-1.15, **p=0.007**)
6/6 to 6/9 (Reference)	52035 (89.4)	6158 (10.6)		-	-
< 6/9 to 6/18	3459 (84.7)	626 (15.3)		0.65 (0.60-0.72, **p<0.001**)	0.60 (0.55-0.66, **p<0.001**)
Worse than 6/18††	683 (78.4)	188 (21.6)		0.43 (0.37-0.51, **p<0.001**)	0.40 (0.34-0.48, **p<0.001**)
**YEARS OF REGISTRATION**					
1 – 5 years (Reference)	28809 (80.9)	6822 (19.1)		-	-
6 – 10 years	22948 (84.8)	4103 (15.2)		1.32 (1.27-1.38, **p<0.001**)	1.13 (1.07-1.20, **p<0.001**)
11 – 15 years	18242 (85.6)	3072 (14.4)		1.41 (1.34-1.47, **p<0.001**)	1.22 (1.12-1.33, **p<0.001**)
16 – 20 years	406 (89.6)	47 (10.4)		2.05 (1.53-2.81, **p<0.001**)	1.94 (1.35-2.89, **p=0.001**)

Observations are for 84,449 individuals.
Abbreviations; OR: Odds ratio, CI: Confidence Interval, PTAL: Public Transport Accessibility Level, IMD: Index of Multiple Deprivation.
* Odds ratios greater than 1 imply greater odds of attendance. † Odds ratios mutually adjusted for all factors shown in the table. †† Variable groups with uptake below the national diabetic eye screening programme uptake goal of ≥75%.
Independent variables with missing data categorised as "Unknown": type of diabetes (2.8%), duration of diabetes (1.4%), distance to screening centre (1.3%), and ethnicity (1.4%).

For peer review only - http://bmjopen.bmj.com/site/about/guidelines.xhtml

DISCUSSION

We found that people of Mixed or Black ethnicity with diabetes show very similar likelihoods of attendance at diabetic eye screening appointments compared with White British people, but that individuals of Asian race were more likely to attend than White British in this large, well organised, sociodemographically diverse urban DESP. This is the most current study with large scale data on ethnicity and diabetic eye screening. In addition, those with poorer visual acuity, younger age and residing in areas with higher levels of deprivation were less likely to attend for diabetic eye screening appointments.

Principal findings and comparison with other studies

Black, Asian and minority ethnic (BAME) groups have been reported to be more likely to develop diabetic retinopathy than White Europeans, more likely to present with sight-threatening retinopathy, (16, 37, 38) and less likely to attend for diabetic eye screening.(15, 16, 24) The commissioning and provision of diabetes eye screening programmes has improved since previous analyses were conducted. The audit was carried out in a large programme with an appointment capacity allowing re-scheduling to meet patients' availability and easily accessible by telephone and email. The cultural and language barriers perceived to prevent older people from BAME groups attending have proved to be misplaced. Attendance rates for BAME groups in our study were all higher than the White British, except for the small Mixed ethnic group, which had a lower, though non-significant, rate of attendance (4.2% uptake difference). Chinese, South Asian and any other Asian background ethnicities were most likely to attend, more so than any other ethnic group. These findings suggest that the underlying increased rates of retinopathy and sight-threatening retinopathy reported in BAME ethnic groups (38, 39) are not explained by non-attendance, raising the issue of increased susceptibility or poorer diabetic control. Although a study by Gulliford et al.(16) analysing sociodemographic inequalities in diabetic eye screening in South London had a high proportion of missing data on ethnicity (~39%), they also reported that African, Caribbean and other ethnicity groups were more likely to attend for diabetic eye screening than White Europeans. Uptake was higher amongst older people and those from BAME groups. All appointment letters are written in English, these data show that the language of the letter was no barrier to better attendance.

Socioeconomic deprivation has consistently been associated with attendance, where those from more deprived areas are less likely to attend for eye screening appointments.(15, 17, 20, 21, 26, 27) Although the overall average difference in attendance of 3.5% between most and least deprived areas found in our study is less than the 9.3% reported in earlier studies,(26) this is still greater than the 2% uptake difference found in a population from South London in 2010.(16) Our results provide further evidence of the ingrained health inequalities present in a multi-ethnic study population with high levels of deprivation. Also, we show the effect of multiple risk factors that appear to impact on attendance. Longer duration of diabetes and worsening visual acuity showed an association with non-attendance compared with individuals with shorter disease duration and better visual acuity. Previous reports have shown an association of longer duration of diabetes with non-attendance.(16, 17) Given that duration of diabetes is one of the three major risk factors for diabetic retinopathy,(4, 40, 41) and considering that >60% of people with type 2 diabetes and almost

For peer review only - http://bmjopen.bmj.com/site/about/guidelines.xhtml

all people with type 1 diabetes will have diabetic retinopathy after 20+ years duration of the disease,(40) the reduced odds of attendance observed in this group, places these patients at increased risk of visual complications. There is, to our knowledge no evidence available about the association of visual acuity with attendance to diabetic eye screening.

In other areas of the UK, increased distance from screening clinic has been associated with an increased risk of non-attendance.(20, 37) We have found that only individuals living ≥ 9 km from a screening centre were formally less likely to attend, but there was evidence of a trend in non-attendance with increasing distance. It is noteworthy that an 8 km radius from one of the NELDESP screening centre covers all of the geographic areas of the 6 CCGs, and it is possible that people living beyond 8km may have moved outside the CCG areas and not updated their GP. Interestingly, we found that the association of distance to screening centre with non-attendance is independent from PTALs in this inner-city population. This may be due to London having a well-developed public transport network and good transport-related access. These findings may not apply elsewhere, particularly to non-urban populations less served by public transport.

In accordance with previous evidence,(10, 15-17, 20, 21, 42, 43) young individuals from 12 to 45 years of age had lower odds of attendance compared with people age 46-60 years. Possible underpinning factors are over confidence about their health or demanding work schedules.(20, 25) Nonetheless, within the context of diabetes chronicity and the need for regular contact with health care services, these individuals are at increased risk of complications through longer duration of disease and possible suboptimal metabolic control.(44)

Our study has several strengths. First, a large sample size with considerable proportions of individuals from different ethnic groups representing a diverse population group all living within the programme area, with one of the most complete datasets on ethnicity reported to date. Second, the use of PTALs in addition to distance to screening centre to evidence the associations of accessibility and transport with attendance. And third, the fact that three quarters of the participants were distributed between two of the most deprived quintiles of IMD, allowing the comparative association between deprivation and ethnicity with attendance to be examined. Our study has several limitations. First, major systemic risk factors for diabetic retinopathy incidence and progression, namely hypertension and glycaemic control, were not available to include in our analysis. Second, we did not analyse the association of the sociodemographic variables with the presence of diabetic retinopathy, which although desirable, would have been difficult to ascertain for repeated non-attenders. Further work to unravel the interplay between ethnicity, deprivation and disease severity, is needed to inform strategies to improve attendance, particularly in high risk under privileged groups.

Conclusion

Smaller previous studies have reported an association between non-white ethnicities and poor attendance at diabetic eye screening appointments, however, in this large diverse urban population, South Asian, Chinese, and individuals of any other Asian background were more likely to attend for diabetic eye screening than White British people. Public health strategies

For peer review only - http://bmjopen.bmj.com/site/about/guidelines.xhtml

have in the past focussed on ethnic differences as a possible cause of variance in diabetic eye screening uptake. The data from this large cohort shows that there are other more influential factors. We have shown that worse visual acuity, higher levels of deprivation, younger age, and longer duration of diabetes are associated with non-attendance. Hence, strategies to improve uptake should be directed at these groups, in order to reduce inequalities in diabetic eye screening.

Footnotes

Contributorship statement: All authors meet the ICMJE criteria for authorship. JA, CE, LB, AT, AR, CGO and AO-B: designed the study. AO-B, MS, TFCH and RC: undertook data management, processing and analysis. AR, CGO and CE provided statistical advice and analysed the data. JA, CE, LB, AR, AO-B wrote the first draft of the report, which was critically appraised by all authors. All the authors read and approved the final draft for journal publication. JA is responsible for data integrity.

Competing interests: None.

Funding: AO-B received a proportion of his funding from the Mexican National Council of Science and Technology (CONACYT, scholarship #2018-000009-01EXTF-00573 to AO-B). CE and AT received a proportion of their salary from the National Institute for Health Research Centre for Ophthalmology at Moorfields Eye Hospital, and UCL Institute of Ophthalmology. The views expressed in the publication are those of the authors and not necessarily those of the Department of Health.

Patient consent for publication: Not required

Ethics approval: This study was registered as an audit and approved through the research governance process at the Homerton University Hospital NHS Foundation Trust and adhered to the UK Data Protection Act 2018.

Transparency statement: The corresponding author affirms that the manuscript is an honest, accurate, and transparent account of the study being reported. No important aspects of the study have been omitted and all discrepancies from the study as planned have been explained.

Data sharing statement: The data that supports the findings of this study are available from the North East London Diabetic Eye Screening Programme upon reasonable request.

For peer review only

References

1. Liew G, Michaelides M, Bunce C. A comparison of the causes of blindness certifications in England and Wales in working age adults (16-64 years), 1999-2000 with 2009-2010. *BMJ Open* 2014;4(2):e004015. doi: 10.1136/bmjopen-2013-004015 [published Online First: 2014/02/15]
2. Solomon SD, Chew E, Duh EJ, et al. Diabetic Retinopathy: A Position Statement by the American Diabetes Association. *Diabetes Care* 2017;40(3):412-18. doi: 10.2337/dc16-2641 [published Online First: 2017/02/23]
3. Diabetes UK. Diabetes prevalence 2019 [Available from: https://www.diabetes.org.uk/professionals/position-statements-reports/statistics/diabetes-prevalence-2019 accessed 27 Feb 2020.
4. Mathur R, Bhaskaran K, Edwards E, et al. Population trends in the 10-year incidence and prevalence of diabetic retinopathy in the UK: a cohort study in the Clinical Practice Research Datalink 2004-2014. *BMJ Open* 2017;7(2):e014444. doi: 10.1136/bmjopen-2016-014444 [published Online First: 2017/03/02]
5. Ferris FL, 3rd. How effective are treatments for diabetic retinopathy? *JAMA* 1993;269(10):1290-1. [published Online First: 1993/03/10]
6. Rohan TE, Frost CD, Wald NJ. Prevention of blindness by screening for diabetic retinopathy: a quantitative assessment. *BMJ* 1989;299(6709):1198-201. doi: 10.1136/bmj.299.6709.1198 [published Online First: 1989/11/11]
7. Scanlon PH. The English National Screening Programme for diabetic retinopathy 2003-2016. *Acta Diabetol* 2017;54(6):515-25. doi: 10.1007/s00592-017-0974-1 [published Online First: 2017/02/23]
8. Public Health England. Diabetic eye screening standards valid for data collected from 1 April 2019 2019 [Available from: https://www.gov.uk/government/publications/diabetic-eye-screening-programme-standards/diabetic-eye-screening-standards-valid-for-data-collected-from-1-april-2019#des-s07-uptake-routine-digital-screening accessed 27 Feb 2020.
9. Public Health England. Diabetic Eye Screening: 2016 to 2017 data 2017 [Available from: https://www.gov.uk/government/publications/diabetic-eye-screening-2016-to-2017-data accessed 01, July 2020.
10. Forster AS, Forbes A, Dodhia H, et al. Non-attendance at diabetic eye screening and risk of sight-threatening diabetic retinopathy: a population-based cohort study. *Diabetologia* 2013;56(10):2187-93. doi: 10.1007/s00125-013-2975-0 [published Online First: 2013/06/25]
11. Virk R, Binns AM, Chambers R, et al. How is the risk of being diagnosed with referable diabetic retinopathy affected by failure to attend diabetes eye screening appointments? *Eye (Lond)* 2020 doi: 10.1038/s41433-020-0877-1 [published Online First: 2020/04/23]
12. Borrell C, Pons-Vigues M, Morrison J, et al. Factors and processes influencing health inequalities in urban areas. *J Epidemiol Community Health* 2013;67(5):389-91. doi: 10.1136/jech-2012-202014 [published Online First: 2013/02/16]
13. Riva M, Curtis S, Gauvin L, et al. Unravelling the extent of inequalities in health across urban and rural areas: evidence from a national sample in England. *Soc Sci Med* 2009;68(4):654-63. doi: 10.1016/j.socscimed.2008.11.024 [published Online First: 2008/12/26]

For peer review only - http://bmjopen.bmj.com/site/about/guidelines.xhtml

14. Dorling D. Editorial—Inequality and Injustice: Some News from Britain. *Urban Geography* 2012;33(5):621-29. doi: 10.2747/0272-3638.33.5.621
15. Millett C, Dodhia H. Diabetes retinopathy screening: audit of equity in participation and selected outcomes in South East London. *J Med Screen* 2006;13(3):152-5. doi: 10.1258/096914106778440608 [published Online First: 2006/09/30]
16. Gulliford MC, Dodhia H, Chamley M, et al. Socio-economic and ethnic inequalities in diabetes retinal screening. *Diabet Med* 2010;27(3):282-8. doi: 10.1111/j.1464-5491.2010.02946.x [published Online First: 2010/06/12]
17. Leese GP, Boyle P, Feng Z, et al. Screening uptake in a well-established diabetic retinopathy screening program: the role of geographical access and deprivation. *Diabetes Care* 2008;31(11):2131-5. doi: 10.2337/dc08-1098 [published Online First: 2008/08/30]
18. Dervan E, Lillis D, Flynn L, et al. Factors that influence the patient uptake of diabetic retinopathy screening. *Ir J Med Sci* 2008;177(4):303-8. doi: 10.1007/s11845-008-0192-5 [published Online First: 2008/07/22]
19. van Eijk KN, Blom JW, Gussekloo J, et al. Diabetic retinopathy screening in patients with diabetes mellitus in primary care: Incentives and barriers to screening attendance. *Diabetes Res Clin Pract* 2012;96(1):10-6. doi: 10.1016/j.diabres.2011.11.003 [published Online First: 2011/12/06]
20. Moreton RBR, Stratton IM, Chave SJ, et al. Factors determining uptake of diabetic retinopathy screening in Oxfordshire. *Diabet Med* 2017;34(7):993-99. doi: 10.1111/dme.13350 [published Online First: 2017/03/16]
21. Orton E, Forbes-Haley A, Tunbridge L, et al. Equity of uptake of a diabetic retinopathy screening programme in a geographically and socio-economically diverse population. *Public Health* 2013;127(9):814-21. doi: 10.1016/j.puhe.2013.04.015 [published Online First: 2013/08/27]
22. Gange WS, Xu BY, Lung K, et al. Rates of Eye Care and Diabetic Eye Disease Among Insured Patients with Newly-diagnosed Type 2 Diabetes. *Ophthalmol Retina* 2020 doi: 10.1016/j.oret.2020.07.004 [published Online First: 2020/07/13]
23. Al-Alawi A, Al-Hassan A, Chauhan D, et al. Knowledge, Attitude, and Perception of Barriers for Eye Care among Diabetic Persons Registered at Employee Health Department of a Tertiary Eye Hospital of Central Saudi Arabia. *Middle East Afr J Ophthalmol* 2016;23(1):71-4. doi: 10.4103/0974-9233.164629 [published Online First: 2016/03/10]
24. Fathy C, Patel S, Sternberg P, Jr., et al. Disparities in Adherence to Screening Guidelines for Diabetic Retinopathy in the United States: A Comprehensive Review and Guide for Future Directions. *Semin Ophthalmol* 2016;31(4):364-77. doi: 10.3109/08820538.2016.1154170 [published Online First: 2016/04/27]
25. Lindenmeyer A, Sturt JA, Hipwell A, et al. Influence of primary care practices on patients' uptake of diabetic retinopathy screening: a qualitative case study. *Br J Gen Pract* 2014;64(625):e484-92. doi: 10.3399/bjgp14X680965 [published Online First: 2014/07/30]
26. Scanlon PH, Carter SC, Foy C, et al. Diabetic retinopathy and socioeconomic deprivation in Gloucestershire. *J Med Screen* 2008;15(3):118-21. doi: 10.1258/jms.2008.008013 [published Online First: 2008/10/18]
27. Waqar S, Bullen G, Chant S, et al. Cost implications, deprivation and geodemographic segmentation analysis of non-attenders (DNA) in an established diabetic retinopathy

For peer review only

For peer review only - http://bmjopen.bmj.com/site/about/guidelines.xhtml

screening programme. *Diabetes Metab Syndr* 2012;6(4):199-202. doi: 10.1016/j.dsx.2012.08.009 [published Online First: 2012/12/04]
28. Public Health England. Screening Quality Assurance Visit Report. NHS Diabetic Eye Screening Service North East London Diabetic Eye Screening Programme. 2017
29. Ministry of Housing Communities & Local Government. The English Indices of Deprivation 2019 (IoD 2019). 2019
30. Pittini M, Rocks C, Leeser R. London geographies and economic performance – insights from the Local Industrial Strategy evidence base interim report. *GLA Economics* 2019(215):12-16.
31. Office of National Statistics. Ethnicity and National Identity in England and Wales: 2011 2011 [Available from: https://www.ons.gov.uk/peoplepopulationandcommunity/culturalidentity/ethnicity/articles/ethnicityandnationalidentityinenglandandwales/2012-12-11#measuring-ethnicity accessed April 13th 2020.
32. Government UK. UK population by ethnicity. Regional Ethnic Diversity 2011 [Available from: https://www.ethnicity-facts-figures.service.gov.uk/uk-population-by-ethnicity/national-and-regional-populations/regional-ethnic-diversity/latest accessed 5 July 2020.
33. Olvera-Barrios A, Heeren TF, Balaskas K, et al. Comparison of true-colour wide-field confocal scanner imaging with standard fundus photography for diabetic retinopathy screening. *Br J Ophthalmol* 2020 doi: 10.1136/bjophthalmol-2019-315269 [published Online First: 2020/03/07]
34. Transport for London. Public Transport Accessibility Levels 2017 [Available from: https://data.london.gov.uk/dataset/public-transport-accessibility-levels#:~:text=The%20method%20is%20essentially%20a,excellent%20access%20to%20public%20transport. accessed 6 July 2020.
35. Transport for London. WebCAT planning tool 2017 [Available from: https://tfl.gov.uk/info-for/urban-planning-and-construction/planning-with-webcat/webcat?intcmp=25932 accessed 6 July 2020.
36. R: A Language and Environment for Statistical Computing [program]. Viena, Austria: R Foundation for Statistical Computing, 2020.
37. Kliner M, Fell G, Gibbons C, et al. Diabetic retinopathy equity profile in a multi-ethnic, deprived population in Northern England. *Eye (Lond)* 2012;26(5):671-7. doi: 10.1038/eye.2012.3 [published Online First: 2012/02/04]
38. Sivaprasad S, Gupta B, Gulliford MC, et al. Ethnic variation in the prevalence of visual impairment in people attending diabetic retinopathy screening in the United Kingdom (DRIVE UK). *PLoS One* 2012;7(6):e39608. doi: 10.1371/journal.pone.0039608 [published Online First: 2012/07/05]
39. Sivaprasad S, Gupta B, Crosby-Nwaobi R, et al. Prevalence of diabetic retinopathy in various ethnic groups: a worldwide perspective. *Surv Ophthalmol* 2012;57(4):347-70. doi: 10.1016/j.survophthal.2012.01.004 [published Online First: 2012/05/01]
40. Fong DS, Aiello L, Gardner TW, et al. Retinopathy in diabetes. *Diabetes Care* 2004;27 Suppl 1:S84-7. doi: 10.2337/diacare.27.2007.s84 [published Online First: 2003/12/25]
41. Yau JW, Rogers SL, Kawasaki R, et al. Global prevalence and major risk factors of diabetic retinopathy. *Diabetes Care* 2012;35(3):556-64. doi: 10.2337/dc11-1909 [published Online First: 2012/02/04]

For peer review only - http://bmjopen.bmj.com/site/about/guidelines.xhtml

42. Scanlon PH, Stratton IM, Leese GP, et al. Screening attendance, age group and diabetic retinopathy level at first screen. *Diabet Med* 2016;33(7):904-11. doi: 10.1111/dme.12957 [published Online First: 2015/09/05]
43. Ahmad B, Neilson F. A Health Equity Audit of the Diabetic Eye Screening Programmes in Cumbria and the North East. *British Journal of Diabetes* 2019;19(1):42-44. doi: 10.15277/bjd.2019.203
44. Hainsworth DP, Bebu I, Aiello LP, et al. Risk Factors for Retinopathy in Type 1 Diabetes: The DCCT/EDIC Study. *Diabetes Care* 2019;42(5):875-82. doi: 10.2337/dc18-2308 [published Online First: 2019/03/06]

For peer review only

STROBE Statement—Checklist of items that should be included in reports of ***cohort studies***

	Item No	Recommendation	Page No
**Title and abstract**	1	(*a*) Indicate the study's design with a commonly used term in the title or the abstract	1-2
		(*b*) Provide in the abstract an informative and balanced summary of what was done and what was found	2
**Introduction**			
Background/rationale	2	Explain the scientific background and rationale for the investigation being reported	4
Objectives	3	State specific objectives, including any prespecified hypotheses	4
**Methods**			
Study design	4	Present key elements of study design early in the paper	5
Setting	5	Describe the setting, locations, and relevant dates, including periods of recruitment, exposure, follow-up, and data collection	5
Participants	6	(*a*) Give the eligibility criteria, and the sources and methods of selection of participants. Describe methods of follow-up	5
		(*b*) For matched studies, give matching criteria and number of exposed and unexposed	NA
Variables	7	Clearly define all outcomes, exposures, predictors, potential confounders, and effect modifiers. Give diagnostic criteria, if applicable	5-6
Data sources/ measurement	8*	For each variable of interest, give sources of data and details of methods of assessment (measurement). Describe comparability of assessment methods if there is more than one group	5-6
Bias	9	Describe any efforts to address potential sources of bias	5-6
Study size	10	Explain how the study size was arrived at	5-6
Quantitative variables	11	Explain how quantitative variables were handled in the analyses. If applicable, describe which groupings were chosen and why	5-6
Statistical methods	12	(*a*) Describe all statistical methods, including those used to control for confounding	6
		(*b*) Describe any methods used to examine subgroups and interactions	6
		(*c*) Explain how missing data were addressed	6
		(*d*) If applicable, explain how loss to follow-up was addressed	NA
		(*e*) Describe any sensitivity analyses	NA
**Results**			
Participants	13*	(a) Report numbers of individuals at each stage of study—eg numbers potentially eligible, examined for eligibility, confirmed eligible, included in the study, completing follow-up, and analysed	7
		(b) Give reasons for non-participation at each stage	7
		(c) Consider use of a flow diagram	7
Descriptive data	14*	(a) Give characteristics of study participants (eg demographic, clinical, social) and information on exposures and potential confounders	7
		(b) Indicate number of participants with missing data for each variable of interest	10
		(c) Summarise follow-up time (eg, average and total amount)	NA
Outcome data	15*	Report numbers of outcome events or summary measures over time	7-10

For peer review only

Main results	16	(*a*) Give unadjusted estimates and, if applicable, confounder-adjusted estimates and their precision (eg, 95% confidence interval). Make clear which confounders were adjusted for and why they were included	7-10
		(*b*) Report category boundaries when continuous variables were categorized	7-10
		(*c*) If relevant, consider translating estimates of relative risk into absolute risk for a meaningful time period	NA
Other analyses	17	Report other analyses done—eg analyses of subgroups and interactions, and sensitivity analyses	7
**Discussion**			
Key results	18	Summarise key results with reference to study objectives	11-12
Limitations	19	Discuss limitations of the study, taking into account sources of potential bias or imprecision. Discuss both direction and magnitude of any potential bias	12
Interpretation	20	Give a cautious overall interpretation of results considering objectives, limitations, multiplicity of analyses, results from similar studies, and other relevant evidence	11-12
Generalisability	21	Discuss the generalisability (external validity) of the study results	11-12
**Other information**			
Funding	22	Give the source of funding and the role of the funders for the present study and, if applicable, for the original study on which the present article is based	13

*Give information separately for exposed and unexposed groups.

Note: An Explanation and Elaboration article discusses each checklist item and gives methodological background and published examples of transparent reporting. The STROBE checklist is best used in conjunction with this article (freely available on the Web sites of PLoS Medicine at http://www.plosmedicine.org/, Annals of Internal Medicine at http://www.annals.org/, and Epidemiology at http://www.epidem.com/). Information on the STROBE Initiative is available at http://www.strobe-statement.org.

For peer review only
